# The Effect of Salt-Tolerant Antagonistic Bacteria CZ-6 on the Rhizosphere Microbial Community of Winter Jujube (*Ziziphus jujuba* Mill. “Dongzao”) in Saline-Alkali Land

**DOI:** 10.1155/2021/5171086

**Published:** 2021-09-24

**Authors:** YanYan Zhou, LiPing Hao, Chao Ji, QiSheng Zhou, Xin Song, Yue Liu, HuYing Li, ChaoHui Li, QiXiong Gao, JinTai Li, PengCheng Zhang, XunLi Liu

**Affiliations:** ^1^College of Forestry, Shandong Agricultural University, Taian, China; ^2^College of Plant Conservation, Shandong Agricultural University, Taian, China; ^3^Xintai Animal Husbandry and Veterinary Service Center, Taian, China; ^4^State Forestry and Grassland Administration Key Laboratory of Silviculture in Downstream Areas of the Yellow River, Shandong Agriculture University, Taian, China

## Abstract

As the main economic crop cultivated in the Yellow River Delta, winter jujube contains various nutrients. However, soil salinization and fungal diseases have affected the yield and quality of winter jujube. In order to use plant growth-promoting rhizobacteria (PGPR) to reduce these damages, the antagonistic bacteria CZ-6 isolated from the rhizosphere of wheat in saline soil was selected for experiment. Gene sequencing analysis identified CZ-6 as *Bacillus amyloliquefaciens*. In order to understand the salt tolerant and disease-resistant effects of CZ-6 strain, determination of related indicators of salt tolerance, pathogen antagonistic tests, and anti-fungal mechanism analyses was carried out. A pot experiment was conducted to evaluate the effect of CZ-6 inoculation on the rhizosphere microbial community of winter jujube. The salt tolerance test showed that CZ-6 strain can survive in a medium with a NaCl concentration of 10% and produces indole acetic acid (IAA) and 1-aminocyclopropane-1-carboxylic acid (ACC) deaminase. Studies on the inhibition mechanism of pathogenic fungi show that CZ-6 can secrete cellulase, protease, and xylanase. Gas chromatography-mass spectrometry (GC-MS) analysis showed that CZ-6 can release volatile organic compounds (VOCs), including 2-heptanone and 2-nonanone. In addition, the strain can colonize the rhizosphere and migrate to the roots, stems, and leaves of winter jujube, which is essential for plant growth or defense against pathogens. Illumina MiSeq sequencing data indicated that, compared to the control, the abundance of salt-tolerant bacteria *Tausonia* in the CZ-6 strain treatment group was significantly increased, while the richness of *Chaetomium* and *Gibberella* pathogens was significantly reduced. Our research shows that CZ-6 has the potential as a biological control agent in saline soil. Plant damage and economic losses caused by pathogenic fungi and salt stress are expected to be alleviated by the addition of salt-tolerant antagonistic bacteria.

## 1. Introduction

Located in the northern part of the Shandong Province, the Yellow River Delta is a newly developed salinization system in China. The salinization of the soil in this area is prominent with a fragile ecological environment, which severely restricts agricultural activities in that region [1]. Winter jujube (*Ziziphus jujuba* Mill. “Dongzao”) is the main economic crop cultivated in the Yellow River Delta [2]. Under field conditions, winter jujube was exposed to salt stress and fungal diseases at the same time. Soil salinization affects the occurrence and spread of pathogens, increasing the invasiveness of plant pathogenic fungi [3]. Common jujube fungal diseases include gray mold, stem rot, and anthracnose, which are usually caused by *Botrytis cinerea*, *Botryosphaeria dothidea*, and *Colletotrichum gloeosporioides*, respectively [4]. Jujube contains a variety of chemical components, including vitamins, polysaccharides, phenols, flavonoids, and nucleosides [5]. However, soil salinization and fungal diseases often cause yield and quality drops.

Plant growth-promoting rhizobacteria (PGPR) play an important role in alleviating salt stress and preventing diseases. PGPR regulate plant hormones by releasing exogenous hormones, metabolites, and enzymes, such as indole acetic acid (IAA), 1-aminocyclopropane-1-carboxylic acid (ACC) deaminase, extracellular polysaccharides, and polyamines, to improve salt tolerance [6]. Bacteria with IAA properties and high ACC deaminase activity are considered effective PGPR [7]. They use ACC as a source of nitrogen and energy, converting it into ammonia and *α*-ketobutyrate to prevent the accumulation of ethylene and strengthen the root system to cope with environmental stress [8]. Inoculation of IAA-producing bacteria can promote the formation of lateral roots and root hairs and can improve the tolerance of plants to salinity [9]. Previous research has indicated that the application of *Bacillus subtilis* GOT9 leads to increased salt stress tolerance of Arabidopsis and effectively reduced the damage of salt stress to plants [10].

On the other hand, using PGPR for biological control is an effective and harmless strategy to reduce the damage caused by plant pathogens to crops [11]. Among them, bacteria are considered instrumental in the development of commercial products [12]. Study has shown that *Bacillus* has a potential antagonistic effect on various plant pathogenic fungi, which is beneficial for plant growth and environmental adaptation [13]. For instance, Chen et al. [14] found that *B. amyloliquefaciens* PG12 was an effective biocontrol agent against apple ring rot, while Jiao et al. [15] found that *B. amyloliquefaciens* YN201732 could be used as a biocontrol agent and plant-growth promoting bacterium in tobacco agriculture. Despite an increasing number of studies pertaining to the biological control of agricultural and forestry crops, there are limited reports on the biological control of winter jujube.

In this study, we report that the salt-tolerant strain CZ-6, isolated from the rhizosphere of wheat, has antagonistic effects on various plant pathogenic fungi. CZ-6 was identified as *B. amyloliquefaciens*. Therefore, the present study is aimed at (1) studying the salt tolerance characteristics of CZ-6 strain, (2) understanding the mechanism underlying the effects of the CZ-6 strain against pathogenic fungi, and (3) exploring the effect of salt-tolerant antagonistic bacteria CZ-6 on the rhizosphere microbial community of winter jujube. These results will provide a theoretical basis for the development and application of salt-tolerant antagonistic CZ-6 as a biological control agent in saline-alkali soils. For farmers, this may be an economically promising alternative to solve the fungal disease of winter jujube in salinized areas.

## 2. Materials and Methods

### 2.1. Sample Collection and Strain Isolation

Soil samples were collected in May 2016 from wheat rhizosphere in the secondary salinization area of the Yellow River Delta, Shandong Province (118° 49′ 15^″^ E, 37° 24′ 31^″^ N). Wheat seedlings were uprooted to keep the root system intact. Excess loose soil is removed by gently shaking, and the soil attached to the roots is considered to be rhizosphere soil [16]. One gram of soil was placed in 100 mL sterile water and mixed on a table concentrator for 30 min. After being serially diluted to 10^−6^-fold, the obtained soil solution was spread on potato dextrose agar (PDA) plates and cultured at 28 ± 2°C for 2-3 days. After purifying isolates, the salt tolerance, ACC deaminase, and IAA production of the isolated strains were determined as follows. The dilution coating method was used to detect the surviving strain in Luria–Bertani (LB) broth with an NaCl concentration of 10% [17]. ACC deaminase production was quantitatively determined using the method described by Penrose and Glick [18]. The ability to produce IAA was determined according to the method described by Bric et al. [19].

### 2.2. Screening and Identification of Strains

In order to obtain bacteria that have inhibitory effects on the pathogenic fungi of winter jujube, the double culture test was used to screen strains with antagonistic effects against *Colletotrichum gloeosporioides*, *Fusarium oxysporum*, *Bipolaris sorokiniana*, and *Botryosphaeria dothidea*. Analysis of bacterial morphology and the physiological and biochemical characteristics was conducted based on the Bergey's Manual of Determinative Bacteriology [20]. The 16S rDNA gene sequencing analysis and gyrB sequencing were performed as previously described [16, 21]. The obtained amplicons were sequenced by a commercial sequencing company (Liuhe Huada Gene Technology Co., Ltd., Beijing, China). The strain with the highest homology to this sequence was selected using the MEGA7.0 software for homology analysis, and the phylogenetic tree of the CZ-6 strain was constructed using the neighboring method.

### 2.3. Analysis of Volatile Organic Compounds (VOCs) and Determination of Antifungal Activity

In order to understand the antibacterial mechanism of CZ-6, based on the methods of Wang et al. [22], the type and relative contents of VOCs produced by antagonistic bacteria were determined and analyzed using gas chromatography-mass spectrometry (GC-MS). Dual-culture confrontation assays were performed to test the inhibitory effect of VOCs produced by CZ-6 on the winter jujube pathogenic fungi, *Colletotrichum gloeosporioides*, *Fusarium oxysporum*, *Bipolaris sorokiniana*, and *Botryosphaeria dothidea* [4, 23]. All pathogens were obtained from the Applied Microbiology Laboratory of the College of Forestry, Shandong Agricultural University. Two solid substrates containing potato dextrose agar (PDA) (15 mL) were selected. The CZ-6 strain was inoculated in the center of the PDA medium plates and evenly distributed, while inactivated bacteria was used as a control. A phytopathogenic fungi with a diameter of 5 mm was placed in the center of another PDA plate. Then, the two substrates were sealed with parafilm (PM996 parafilm) and incubated at a constant temperature of 30°C in an incubator. According to the description of Sharifi and Ryu [24], the following formula is used to calculate the inhibition rate of pathogenic fungi:

(1) $$\begin{array}{c} \text{inhibition }\left(\%\right)=1-\left(\text{fungal growth}/\text{control growth}\right)\times 100\%. \end{array}$$

### 2.4. Determination of Extracellular Hydrolase

#### 2.4.1. Qualitative Determination

Cellulase, protease, and xylanase were qualitatively and quantitatively measured to further understand whether extracellular hydrolase is involved in inhibiting fungal growth. The qualitative determination of extracellular hydrolase was obtained through the plate diffusion method [25]. Different substrates were added to the solid medium, and the formation of a clear halo around the bacterial colonies was considered to be a positive result of the respective enzyme activity [22]. The ability of proteolytic enzyme production was assessed by inoculating a pure colony of the CZ-6 strain on skim milk agar plates using the method described by Maurhofer et al. [26]. Cellulase activity was examined on carboxymethyl cellulose- (CMC-) agar plates [27], and xylanase activity was assessed by growing bacteria on xylan plates. Plates were incubated at 28 ± 2°C for 5 days.

#### 2.4.2. Quantitative Determination

In order to quantitatively estimate cellulase, protease, and xylanase activities, referring to the method of Wang et al. [22], CZ-6 strains were inoculated into fermentation media with different functions and cultured for 2-3 days. The fermentation broth was centrifuged at 1,073 × *g* for 10 min to prepare a crude enzyme solution, and the activity of the three extracellular hydrolases was quantitatively determined by spectrophotometry. Cellulase and xylanase activity was determined based on the chromogenic reaction of reducing sugar with a color reagent (dinitrosalicylic acid solution) [28]. A Folin–Ciocalteu's phenol reagent was used to determine the protease activity. One unit (U) of the cellulase, protease, and xylanase activities is defined as the amount of enzyme liberating 1 *μ*mol equivalent of glucose, tyrosine, and xylose from sodium carboxymethyl cellulose, casein, and xylan, respectively, per minute [29].

### 2.5. Colonization Characteristics of the CZ-6 Strain

#### 2.5.1. *Cultivation and Inoculation of Mutant Strain*s

In order to facilitate the recovery of strains and verify the identity of the recovered strains, the CZ-6 strain was first induced with rifampicin and spectinomycin to obtain a mutant of the strain. Mutations were described previously [30]. The mutant strain was cultured overnight in LB broth at 30°C and 200 rev min^−1^ to 1 × 10^8^ cfu mL^−1^. The bacterial suspension was centrifuged at 1073 × g for 10 min. The pellet was resuspended in sterile water and adjusted to 4 × 10^8^ cfu mL^−1^. Twenty milliliters of bacterial suspension was poured into the roots of three-year-old winter jujube seedlings [22].

#### 2.5.2. Recovery of Colonizing Strains

The soil was recovered from the rhizosphere of winter jujube every 10 days in order to verify whether the selected strain could colonize the rhizosphere of winter jujube, and the bacterial colonies of the rhizome of winter jujube were counted by the plate dilution method. To evaluate the colonization characteristics of the CZ-6 strain inside the plant, sterilized scissors and tweezers were used to collect the roots, stems, and leaves of the potted winter jujube plants in the greenhouse. The sample tissue was soaked in 70% ethanol for 3 min, treated with 3% sodium hypochlorite for 5 min, then soaked in 70% ethanol for 1 min to sterilize the tissue surface, rinsed with sterile water three times, and dried on sterile filter paper [31]. A sterile blade was then used to cut the plant tissue into fragments of equal size, which were placed on a PDA plate containing 300 *μ*g mL^−1^ rifampicin and spectinomycin and incubated at 30°C for 24 h. The production of colonies in the plant tissues of the control and treatment groups was assessed before being stored on the PDA plates.

#### 2.5.3. DNA Extraction of Rifampin- and Spectinomycin-Resistant Strains

The DNA of rifampin- and spectinomycin-resistant strains was extracted from the rhizosphere soil, roots, stems, and leaves of winter jujube, as described below. These genetic fingerprints were compared with the mutant strain by Rep-PCR to determine whether they were present in the rhizosphere, root, stem, and leaf tissues of winter jujube. The box-air primer (3′-CTACGGCAAGGCGACGCTGACG-5′) was used for Rep-PCR [17].

### 2.6. Determination of Rhizosphere Microbial Diversity and Community Structure of Winter Jujube

#### 2.6.1. Bacteria Inoculation and Rhizosphere Soil Collection

In order to understand the effect of CZ-6 strain inoculation on the rhizosphere microbial community of winter jujube, healthy and uniformly growing three-year-old jujube seedlings were selected for the pot experiment. The bacterial suspension was prepared as described before. After germination, the winter jujube was inoculated by root irrigation. The control group was inoculated with an equal number of inactivated bacteria, and conventional irrigation was used throughout the growth process. Each treatment was repeated three times, with only one plant per pot. Five months after the growth of the winter jujube seedlings, the roots were gently shaken to remove larger soil particles, and the rhizosphere soil tightly attached to the root surface was collected [22]. The control and treatment groups were repeated three times. The soil sample was passed through a 2 mm sieve and thoroughly mixed, before the homogenized samples were stored at -80°C for subsequent analysis of soil microbial community structure.

#### 2.6.2. DNA Extraction and PCR Amplification

Microbial genomic DNA was extracted from each collected soil sample using a Soil DNA kit (TransGen Biotech, Beijing, China) following the manufacturer's protocol. The universal primers, U515F (5′-barcode-GTGCCAGCMGCCGCGG-3′) and U907R (5′-CCGTCAATTCMTTTRAGTTT-3′), were used to amplify the V4 and V5 regions of the 16S rRNA gene from the extracted total bacterial DNA samples to obtain the best possible taxonomic resolution. ITS1F (5′- -barcode-CTTGGTCATTTAGAGGAAGTAA-3′) and 2043R (5′-GCT-GCGTTCTTCATCGATGC-3′) were used to amplify the ITSr DNA gene from the fungal genomic DNA.

#### 2.6.3. Illumina MiSeq Sequencing and Sequence Analysis

To determine the effect of the CZ-6 strain on the rhizosphere microbial community structure of winter jujube, Illumina MiSeq sequencing was used to study the difference between the rhizosphere microbial community of winter jujube uninoculated and inoculated with the CZ-6 strain. High-throughput sequencing of 16S rRNA genes and ITS sequences was performed on an Illumina MiSeq platform (Illumina, USA) by Majorbio Bio-pharm Technology Co., Ltd. (Shanghai, China). QIIME V1.9.1 was used to perform quality control filtering on the quality of reads and splicing effects. Chimera sequences were identified and removed using UCHIME V 7.1. The sequences retained for each sample were subjected to operational taxonomic unit (OTU) cluster analysis using the established UPARSE software [32]. The short, ambiguous, and low-quality reads were removed based on 97% sequence similarity. Finally, the representative sequences of each OTU were classified against the Silva (SSU123) 16S rRNA database for bacteria and the UNITE 7.0/ITS database for fungi using the RDP classifier with a 70% confidence threshold. We estimated fungal and bacterial richness using the Chao and Ace indices [33]. Shannon and Simpson indices were calculated to evaluate species diversity [34].

#### 2.6.4. Statistical Analysis

All experiments were performed in triplicate, and all statistical analyses were performed using the SAS version 8.0 software (SAS Institute, Inc.). Data were analyzed using analysis of variance (ANOVA) using the SAS Software, and significant differences in the diversity and richness indexes between the treatment and the control group were identified using Duncan's multiple-range tests (DMRT). Differences between fungi on the genus level in control group and CZ-6 inoculation were assessed using two-tailed Student's *t*-tests. Differences in mean values were considered significant at *P* < 0.05.

## 3. Results

### 3.1. Screening and Identification of Strains

**Figure 1:**
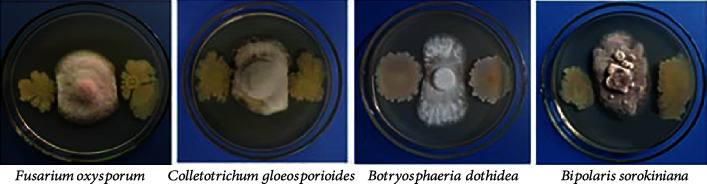
Inhibitory effect of CZ-6 strain on pathogenic fungi.

**Figure 2:**
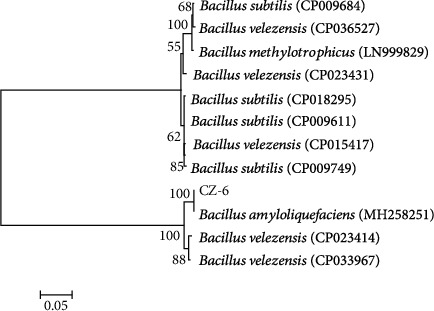
Phylogenetic tree constructed by neighbor joining method. Bootstrap values are indicated on nodes.

The bacteria isolated from the rhizosphere of wheat were subjected to a double culture test to screen for bacteria with inhibitory effects on the fungal pathogens of jujube. The screening results show that CZ-6 has antagonistic effects against *Colletotrichum gloeosporioides*, *Fusarium oxysporum*, *Bipolaris sorokiniana*, and *Botryosphaeria dothidea* (Figure 1). The bacterial colony of strain CZ-6 was light yellow and opaque, moist on the surface, regular at the periphery, and folded in the center (Supplementary Figure S1a). The cells of strain CZ-6 were identified as gram-positive, rod-shaped, and spore-forming (Supplementary Figure S1b). Supplementary Table S1 provides the physiological and biochemical indicators of the CZ-6 strain. Homology analysis of the gyrB sequence revealed that the similarity coefficient between the CZ-6 strain and *B. amyloliquefaciens* was 99% (Figure 2). Subsequently, phylogenetic analysis based on the 16S rDNA sequence also identified it as *B. amyloliquefaciens*. Its sequence was stored in the National Center for Biotechnology Information (NCBI) with the accession number MW165777.1. Determination of related indicators of salt tolerance confirmed that the CZ-6 can survive in 10% NaCl. The ACC deaminase was produced by CZ-6 at a rate of 9.79 ± 0.79 *μ*mol mg^−1^ h^−1^ and IAA at a rate of 28.32 ± 2.67 *μ*g mL^−1^.

### 3.2. Antifungal Activity and Composition of VOCs

**Figure 3:**
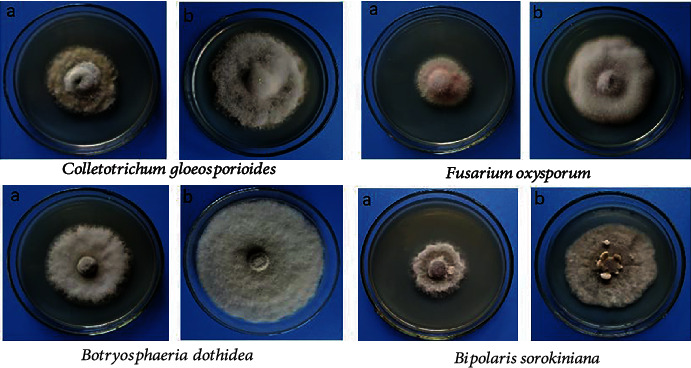
Inhibitory effect of VOCs produced by the CZ-6 strain on pathogenic fungi: (a) treatment group; (b) control group.

We used GC-MS to analyze the VOCs produced by CZ-6 and determine its antifungal activity. The results show that the volatiles produced by the CZ-6 strain growing on PDA medium inhibited a broad range of pathogens (Figure 3). The colony diameter of pathogenic fungi in the treatment and control groups was measured and the inhibition rate calculated. The inhibition of mycelial growth of the volatile metabolites produced by the CZ-6 strain against *C. gloeosporioides*, *F. oxysporum*, *B. sorokiniana*, and *B. dothidea* was 38%, 61%, 38%, and 59%, respectively. The types and relative contents of VOCs produced by the CZ-6 strain were analyzed using GC-MS technology.  Table 1 lists the compounds with a relative peak area greater than 1%, among which 2-methylpropylhydrazine, 2-heptanone, and 2-nonanone were the top three major VOCs.

### 3.3. Extracellular Hydrolase Activity of the CZ-6 Strain

The extracellular hydrolase activity of the CZ-6 strain was evaluated qualitatively and quantitatively in vitro. Qualitative analysis shows that after incubating at 28 ± 2°C for three days, clear and visible dissolution halos formed around the CZ-6 colonies grown on carboxymethyl cellulose agar plates and skimmed milk agar plates (Supplementary Figure S2). This suggests that CZ-6 could produce enzymes capable of degrading fungal cell walls. The quantitative analysis of enzyme production showed that the levels of cellulase and protease reached 10.8 ± 1.23 U mL^−1^ and 653.96 ± 13.72 U mL^−1^, respectively. Interestingly, the level of xylanase secreted by the CZ-6 strain reached 163.35 ± 7.04 U mL^−1^, although no circular hyaline zone formed around the colonies on the plate containing xylan.

### 3.4. Colonization Characteristics

**Figure 4:**
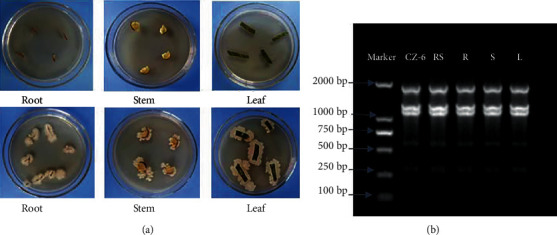
(a) The recovery result of the double antibiotic plate. The samples on the top line and on the bottom line are from uninoculated and inoculated plants, respectively. (b) The 1% agarose gel electrophoresis result of the recovered colony genome. M: Trans2K Trans DNA Marker; CZ-6: strain; RS: rhizosphere; R: root; S: stem; L: leaf.

The pot test was used to determine the colonization ability of the CZ-6 strain. Quantitative analysis showed after 50 days of inoculation; the population density of CZ-6 in the rhizosphere of winter jujube was 4.24 × 10^6^ cfu g^−1^ dry weight of soil. This indicated *B. amyloliquefaciens* CZ-6 could colonize the rhizosphere of the winter jujube. The double antibiotic labeling recovery method showed that the CZ-6 strain was found in the rhizosphere soil, roots, stems, and leaves of winter jujube (Figure 4(a)). In addition, a single bacterial colony on the plate was compared to the CZ-6 strain for BOX-PCR gel electrophoresis. The CZ-6 strain and the isolated bacteria from rhizosphere soil, roots, stems, and leaves showed the same five bands between 250 bp and 2,000 bp in the gel imager (Figure 4(b)). The results showed that the strains isolated from rhizosphere soil, roots, stems, and leaves were all CZ-6 strains. The CZ-6 strain could colonize the rhizosphere of winter jujube and migrate to the roots, stems, and leaves.

### 3.5. Microbial Community Structure after CZ-6 Inoculation

After sequence optimization of the sequencing results, a total of 27,262 16S rDNA effective sequences and 52,264 ITS effective sequences were obtained during the sequence optimization process. Using a 3% difference cutoff, these sequences were divided into 694 fungal OTUs and 3,515 bacterial OTUs. Regardless of whether CZ-6 was inoculated or not, the Shannon, Ace, and Chao indices of bacteria were higher than those of fungi, but the Simpson index was the opposite. The richness index (Ace and Chao) and diversity index (Shannon and Simpson) of the bacterial community of the treatment group inoculated with the CZ-6 strain were not significantly different to the control level. Concerning the fungi, the Shannon index of the fungal community in the treatment group was significantly lower than that of the control group, and the Simpson index was significantly higher than that of the control group. Inoculation with the CZ-6 strain reduced the abundance of fungal communities, but the difference was not significant (Table 2). The above results indicate that following colonization by strain CZ-6, the diversity and richness of the rhizosphere bacterial community of winter jujube did not change significantly, but the fungal community diversity was significantly affected.

### 3.6. Microbial Community Composition after CZ-6 Inoculation

**Figure 5:**
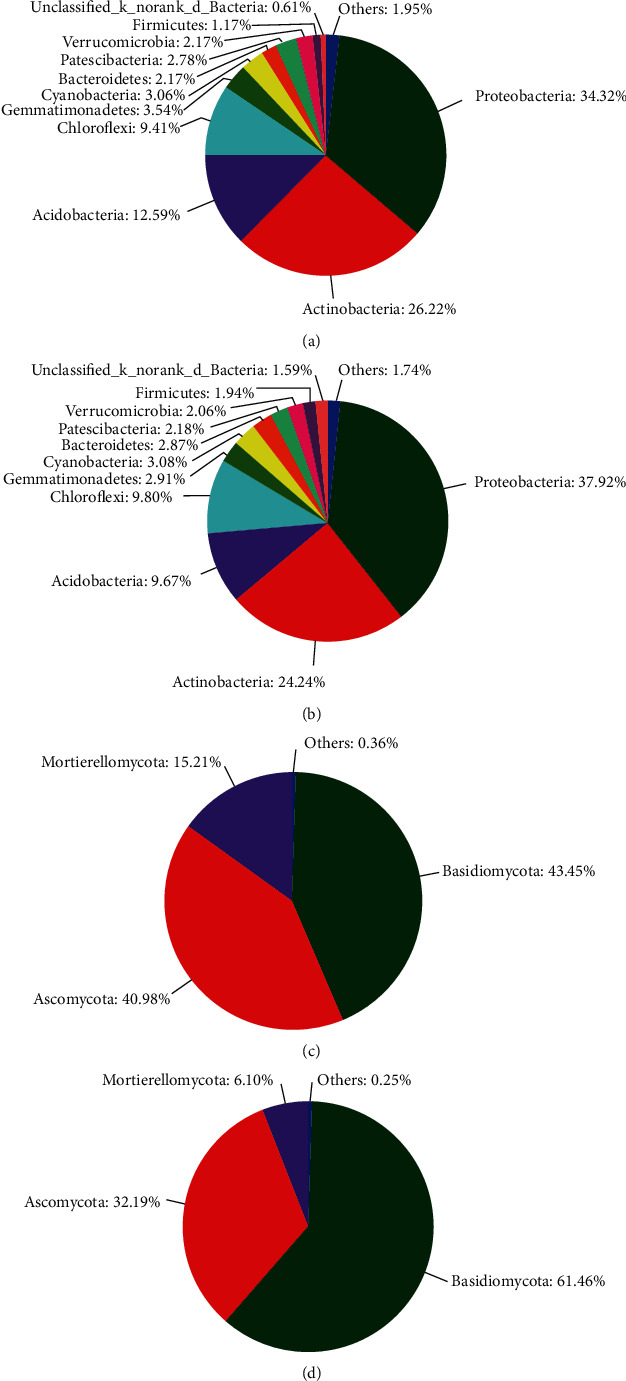
The relative abundance of bacterial phyla (a) in the control groups and (b) in the CZ-6 treatment groups; the relative abundance of fungal phyla (c) in the control groups and (d) in the CZ-6 treatment groups.

**Figure 6:**
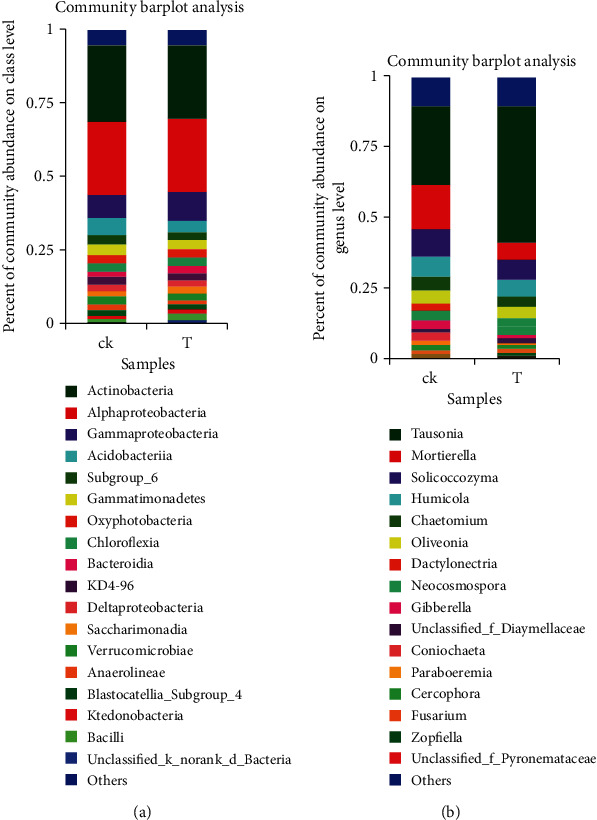
Relative abundance of bacteria on the order level (a) and fungi on genus level (b) from CZ-6 treatment and control groups. CK: control; T: treatment.

**Figure 7:**
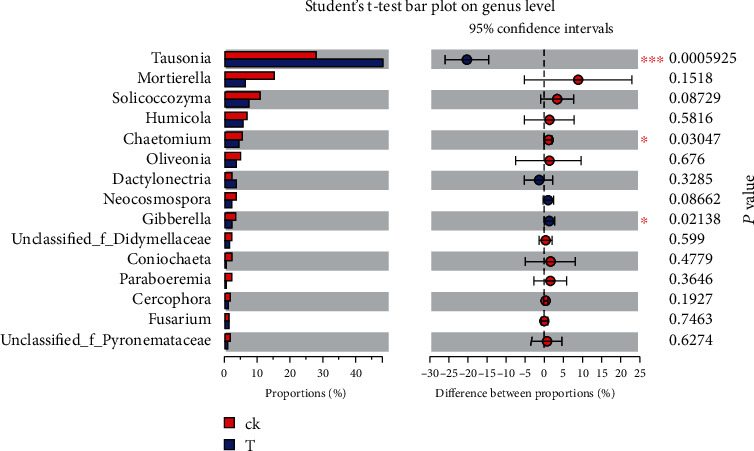
Multifungal genera differences between the treatment groups. CK: control; T: treatment.

The microbial communities of the control and treatment groups were compared to determine the effect of CZ-6 inoculation on the rhizosphere microbial community of winter jujube. The results show that Basidiomycota and Ascomycota were the dominant fungi in the fungal community (Figures 5(c) and 5(d)). Compared to the control, the relative abundance of Basidiomycota in CZ-6-inoculated plants increased by 41.5%; in contrast, the relative abundance of Ascomycota decreased by 27.3% (*P* < 0.05). For bacteria, the top four phyla with the highest total abundance in all samples were Proteobacteria, Actinobacteria, Acidobacteria, and Chloroflexi (Figures 5(a) and 5(b)). The level composition and relative abundance of bacteria in the control and treatment groups were similar, indicating that inoculation of the fermentation broth of the CZ-6 strain had no significant effect on the rhizosphere bacterial community structure of winter jujube (Figure 6(a)). Compared with the control group, the treatment group inoculated with the CZ-6 strain had the same level of fungal species, but the relative abundance was significantly different (Figures 6(b) and 7). In the treatment group, the relative abundance of the dominant genus *Tausonia* increased significantly, and the relative abundance of pathogenic fungi *Chaetomium* and *Gibberella* decreased significantly. The relative content of some pathogenic fungi such as *Mortierella*, *Humicola*, and *Neocosmospora* was lower than that of the control group, but the difference was not significant (Figure 7).

## 4. Discussion

### 4.1. Stress Resistance Characteristics of Isolates

Microorganisms are a source of new compounds with medicinal and agricultural applications [11]. PGPR are effective as stress mitigators, and they show relatively better improvement in growth and yield as well as oxidation parameters of the salt-affected plants [40]. Studies have shown that inoculation with PGPR can promote the growth of wheat and maize in saline-alkali soils and reduce the damage caused by salt stress [17, 41]. Using PGPR for biological control can replace chemical pesticides and manage plant diseases caused by various crop pathogens [42]. Bacteria, such as *Pseudomonas*, *Bacillus*, and *Polymyxa*, are effective in mitigating plant diseases caused by plant pathogens [11, 43]. The strain CZ-6, isolated from saline soil and has broad-spectrum antagonistic activity against a variety of plant pathogens, was selected and identified as *B. amyloliquefaciens* in this study.

In a stress environment, PGPR can produce IAA and ACC deaminase to effectively protect plants [44, 45]. With the increase of environmental stress, the pressure of ethylene on plant growth increases. As a precursor of ethylene, excess ACC is decomposed by PGPR, containing ACC deaminase, resulting in a decrease in ethylene level and limited plant damage [46]. IAA can induce cell elongation and significantly enhance the formation of lateral roots and root hairs [47]. The CZ-6 strain can survive in a medium with 10% NaCl and can secrete ACC deaminase and IAA at the same time, illustrating its potential as a biological inoculum to alleviate plant salt stress.

### 4.2. Mechanisms of Bacillus Controlling Plant Diseases

*Bacillus*, as one of the largest bacterial genera, has been widely used in agricultural biological control due to its strong potential for biological management of various plant diseases [48]. The mechanism by which *Bacillus* spp. control plant diseases includes the lysis of pathogenic fungus hyphae and the production of antifungal metabolites, plant growth promotion (PGP), production of antibiotics, nutrient and space competition, and induction of plant system resistance [49]. In this study, the CZ-6 strain produced several extracellular hydrolases, including cellulase, protease, and xylanase. These enzymes can effectively hydrolyze the main components of fungal cell walls and play an important role in the cell wall lysis of pathogens [50]. The xylanase activity is inconsistent in qualitative and quantitative experiments, which may be related to the inconsistency of the culture medium used in the experiment [16]. Study has shown that the protease produced by *B*. *amyloliquefaciens* has a biological control effect on *Fusarium oxysporum* [51]. The activity intensity of hydrolytic enzymes (protease, cellulase) is the key factor for *Bacillus velezensis* to control *Botrytis cinerea* [52]. Therefore, the strong activity of the hydrolase secreted by *B. amyloliquefaciens* CZ-6 that can dissolve fungal cell walls is consistent with the growth inhibition of a variety of pathogens.

The production of VOCs is another mechanism through which *Bacillus* confers protection to plants [53]. Through GC-MS, a common technique for identifying VOCs present in secondary metabolites [54], 72 VOCs were identified. Similar to some *Bacillus* that produce multiple VOCs [16, 55], the strain CZ-6 also produces VOCs, presumably for biological control. The most volatile compound produced by CZ-6 is 2-methylpropylhydrazine. Although no studies have shown that it antagonizes pathogens, its derivatives can be synthesized into pesticides, suggesting the strain's potential use as microbial pesticide [56]. Recent study has shown that the main VOC produced by the CZ-6 strain, 2-nonanone, can inhibit anthracnose fungus, *Candida*, and *Staphylococcus* [57]; 2-heptanone has also been reported to have strong antifungal activity. The two volatile compounds play an important role in the prevention and control of watermelon wilt [58]. The prerequisite for selecting a biological control agent is that it can inhibit pathogenic fungi and reduce infection, as well as effectively colonize the host and quickly adapt to the surrounding environment [59]. Study has shown that *Bacillus subtilis* B26 can colonize plant roots, stems, and leaves, while increasing the biochemical indicators of plant drought tolerance and alleviating the effects of drought stress [60]. In this study, we found that the CZ-6 strain can colonize the rhizosphere of winter jujube and migrate to the roots, stems, and leaves, indicating that the bacteria can settle and migrate systematically in winter jujube. This provides further insights into the prevention and treatment of pathogens.

### 4.3. Effect of Inoculation on Rhizosphere Microbial Community

Illumina MiSeq sequence analysis showed that inoculation with the CZ-6 strain reduced the diversity and richness of soil fungal communities. At the phylum level, the relative abundance of Basidiomycota increased after inoculation with the CZ-6 strain, which is similar to previous findings [61]. Basidiomycota can form ectomycorrhizas to help plants obtain mineral nutrients from the soil, while plants provide sugar in return [62]. Soil pH and organic matter content are positively correlated with the relative abundance of Ascomycota, so we speculate that CZ-6 inoculation may contribute to the differences in soil physical and chemical properties [63]. At the genus level, compared with the control, except for the significant increase in the dominant genus *Tausonia*, most of the fungal genera showed a downward trend, among which the relative abundance of *Chaetomium* and *Gibberella* was significantly reduced. At present, there are few studies on *Tausonia*. *Tausonia* can grow in the presence of high concentrations of NaCl, and we suspect that the significant increase in its relative abundance increases the number of salt-tolerant microorganisms in the soil [64]. *Tausonia* also has a variety of extracellular hydrolase activities, which can dissolve the cell walls of pathogenic fungi [65]. Rice Bakanae disease, caused by *Gibberella*, is one of the most important seed-borne fungal diseases. *Gibberella* infection reduces seed germination and slows seedling growth [66, 67]. In terms of bacterial community composition, the relative abundance of Alphaproteobacteria and Gammaproteobacteria increased, while Actinobacteria and Acidobacteria decreased. Alphaproteobacteria are involved in biological nitrogen fixation; a large number of nitrogen fixing bacteria in soils and in symbioses with plants are Alphaproteobacteria [68, 69]. Gammaproteobacteria are often isolated in salt water, and their increase may improve the soil's stress tolerance [70]. pH is an important factor driving the composition of bacterial communities, and the decrease of Ascomycota may be related to changes in soil pH [71]. The decrease in the abundance of Acidobacteria may be due to the increase in soil nitrogen content after CZ-6 inoculation, as the abundance of acid bacteria is negatively correlated with nitrogen content [72].

In conclusion, the CZ-6 strain with broad-spectrum antagonistic activity isolated from saline-alkali land was identified as *B. amyloliquefaciens*. This study describes the salt-tolerant properties of *B. amyloliquefaciens* CZ-6 and its mechanism of action as an inhibitor of pathogenic fungi through the production of extracellular hydrolases, the release of volatile compounds, and the effect on pathogens in the jujube rhizosphere soil. The CZ-6 strain has several beneficial effects and may be developed and commercialized for microbial preparation. To the best of our knowledge, this is the first study to investigate the influence of salt-tolerant antagonistic bacteria on the rhizosphere microbial community of crops in saline-alkali soil.

## Figures and Tables

**Table 1:** Volatile organic compounds produced by the CZ-6 strain and their functions.

Serial number	Rt (min)	Components	Area (%)	PGP trait	Reference
2	4.647	2-Imidazolidinone	1.12		Not reported
3	6.242	Silanediol, dimethyl-	1.18		Not reported
4	6.684	Hydrazine, (2-methylpropyl)-	39.52		Not reported
6	7.669	3,4-Hexanediol, 2,5-dimethyl-	1.02		Not reported
8	8.293	2,3-Butanediol, [R-(R^∗^,R^∗^)]-	4.17	Growth-promoting	[35]
13	9.89	Carbonic acid, monoamide	4.51		Not reported
18	10.962	2-Heptanone	5.61	Antagonism	[36]
34	14.701	2-Nonanone	4.82	Antagonism	[37]
35	14.889	2-Nonanol	2.49	Antagonism	Patent (CN201510866778.5)
42	16.906	2-Dodecanone	2.33	Antagonism	Patent (CN201510866778.5)
50	19.45	2-Undecanone	2.39	Nematocidal activities	[38]
51	19.574	2-Pentadecanol	1.12		Not reported
63	23.14	2-Tridecanone	1.21	Antagonism	[39]

**Table 2:** Diversity and richness indices of the bacterial and fungal community from CZ-6 treatment and control groups.

Index	Bacteria		Fungi	
	CK	CZ-6	CK	CZ-6
Shannon	5.92 ± 0.18a	5.95 ± 0.21a	2.79 ± 0.08b	2.34 ± 0.13a
Simpson	0.007 ± 0.002a	0.007 ± 0.002a	0.12 ± 0.01b	0.25 ± 0.03a
Ace	1,578.18 ± 36.26a	1,475.94 ± 126.45a	176.56 ± 12.43a	165.64 ± 24.48a
Chao	1,559.80 ± 48.01a	1,469.12 ± 109.98a	175.78 ± 11.38a	164.50 ± 23.97a
Coverage	0.9896	0.9900	0.9996	0.9997

Values are means ± SD (*n* = 3). Means sharing a common letter within the same column are not significantly different at *P* < 0.05.

## Data Availability

All data generated or analyzed during this study are included in this published article.

## Supplementary Materials

Supplementary 1 Figure S1: colony and spore morphology of CZ-6 strain. Figure S2: qualitative test results of extracellular hydrolase produced by CZ-6 strain. Figure S3: identification of VOCs produced by *B. amyloliquefaciens* CZ-6 by GC-MS.

Supplementary 2 Table S1: physiological and biochemical characteristics of the CZ-6 strain.
