# Supplementary material for: The Effect of Salt-Tolerant Antagonistic Bacteria CZ-6 on the Rhizosphere Microbial Community of Winter Jujube (Ziziphus jujuba Mill. “Dongzao”) in Saline-Alkali Land
Source: Biomed Res Int. 2021 Sep 24;2021:5171086. doi: 10.1155/2021/5171086 (PMC8487612; doi:10.1155/2021/5171086)
Supplement: Supplementary 1 — Figure S1: colony and spore morphology of CZ-6 strain. Figure S2: qualitative test results of extracellular hydrolase produced by CZ-6 strain. Figure S3: identification of VOCs produced by B. amyloliquefaciens CZ-6 by GC-MS. [file 5171086.f1.docx]

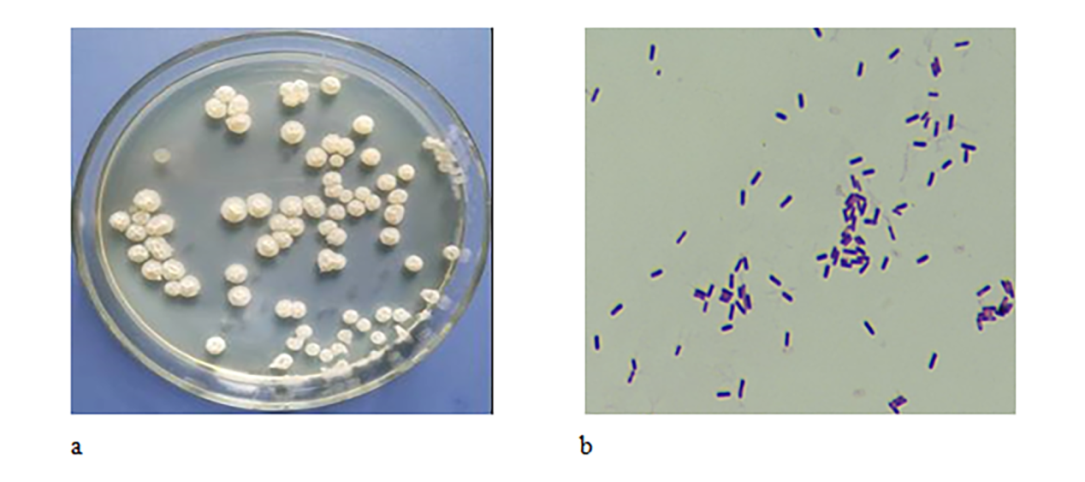
**Figure S1.** Colony and spore morphology of CZ-6 strain. (a) Colony morphology of CZ-6 cultured on LB plates for 24 h; (b) Morphology of bacteria and spores of the CZ-6 strain after 18 h of culture.


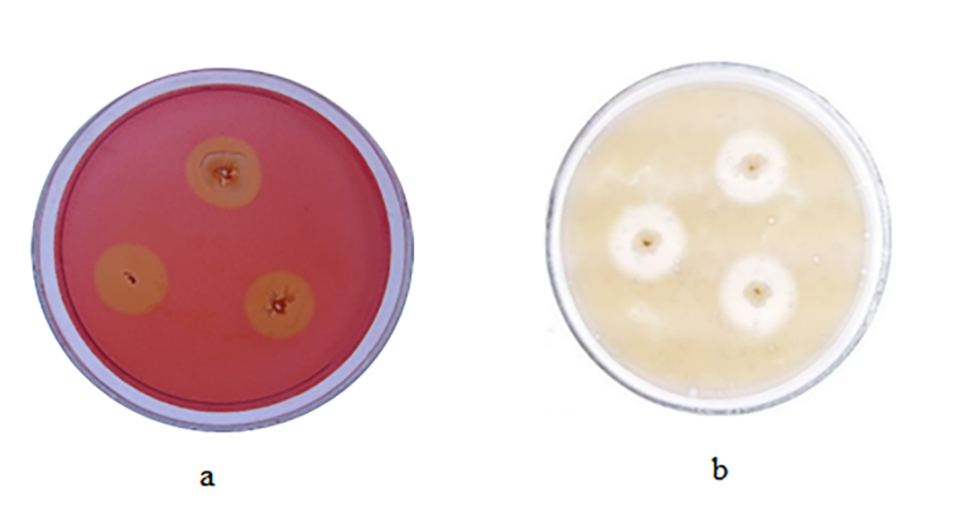
**Figure S2.** Qualitative test results of extracellular hydrolase produced by CZ-6 strain. (a) Cellulase activity was analyzed using carboxyl methyl cellulose agar plates. (b) Protease activity was analyzed using skim milk agar plates.

**Figure S3.** Identification of VOCs produced by *B. amyloliquefaciens* CZ-6 by GC-MS.**
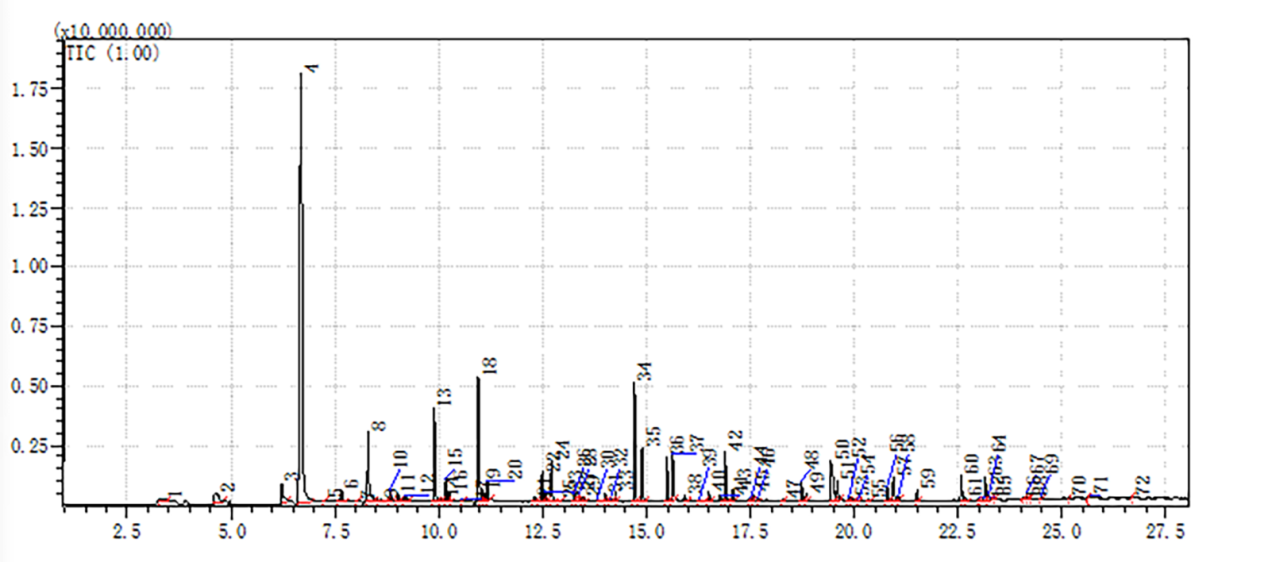
**
