# Supplementary material for: The Effect of Salt-Tolerant Antagonistic Bacteria CZ-6 on the Rhizosphere Microbial Community of Winter Jujube (Ziziphus jujuba Mill. “Dongzao”) in Saline-Alkali Land
Source: Biomed Res Int. 2021 Sep 24;2021:5171086. doi: 10.1155/2021/5171086 (PMC8487612; doi:10.1155/2021/5171086)
Supplement: Supplementary 2 — Table S1: physiological and biochemical characteristics of the CZ-6 strain. [file 5171086.f2.docx]

**Table S1.** Physiological and biochemical characteristics of the CZ-6 strain

| Physiological and biochemical characteristics | Result |
| --- | --- |
| oxidase  contact enzyme | -  + |
| oxygen demand | - |
| H_2_S test | + |
| citrate utilization | + |
| propionate utilization | - |
| phenylalanine amino acid deaminase | - |
| starch hydrolysis | + |
| urea hydrolysis | + |
| methyl red | - |
| V-P test | - |
| nitrate reduction | + |
| tyrosine hydrolysis | - |
| gelatin liquefaction | + |

+ Positive for production; - negative for production.
